# The effect of high-intensity laser therapy on pain and lower extremity function in patellofemoral pain syndrome: a single-blind randomized controlled trial

**DOI:** 10.1007/s10103-024-04017-y

**Published:** 2024-04-17

**Authors:** Ozge Ozlu, Esra Atilgan

**Affiliations:** 1 Department of Physiotherapy and Rehabilitation, Institute of Health Sciences, Istanbul Medipol University, 34815 Istanbul, Turkey; 2 Department of Orthotics and Prosthetic, Institute of Health Sciences, Istanbul Medipol University, Istanbul, Turkey

**Keywords:** Laser therapy, Patellofemoral pain syndrome (PFPS), Range of motion, Quadriceps muscle, Pain

## Abstract

Patellofemoral pain syndrome (PFPS) is a set of symptoms that negatively affect the daily life activities of the individual, leading to functional disability and significant loss of labor, especially in young adults. PFPS is usually due to weakness of the vastus medialis obliquus (VMO) resulting in abnormal patellar tracking and pain. Our study aims to compare the efficacy of high-intensity laser therapy (HILT) on pain and lower extremity function in the treatment of PFPS with different electrophysical agents (EPAs). The study was designed as a single-blind randomized controlled trial. Forty-five people with PFPS (aged 25–45 years) were included in the study. The patients were randomly divided into three groups and a total of ten sessions of treatment were administered to all three groups for 2 weeks, 5 days a week. High-intensity laser (HILT) and exercise program were applied to group 1. Ultrasound (US), transcutaneous electrical nerve stimulation (TENS), and exercise program were applied to group 2. In group 3, US, interferential current (IFC), and exercise program were applied. Both groups underwent three evaluations: pre-treatment, post-treatment, and 12 weeks after treatment. Outcome measures included the visual analog scale for pain severity (VAS), knee flexion range of motion (FROM), Q angle, pain threshold, muscle strength of quadriceps and hamstring, Kujala patellofemoral scoring, lower extremity functional scale (LEFS), and Timed Up and Go Test (TUG). The ANOVA was used for comparing the data of the groups, and two-way repeated measure ANOVA was used to compare at the pre-post and post-intervention 3rd month. The LSD and Bonferroni post hoc tests were also used to identify the between-group differences. Groups 2 and 3 were statistically effective in pain and functionality (*p* < 0.05). Group 1 was found to be statistically more effective than other groups in reducing pain (95% confidence interval (CI), 0.000/0.000; *p* = 0.000), increasing knee flexion angle (95% CI, 127.524/135.809; *p* = 0.000), and increasing lower extremity function (95% CI, 75.970/79.362; *p* = 0.000). This study indicated that high-intensity laser therapy was found to be a more effective method in the treatment of patellofemoral pain syndrome after 3 months of follow-up compared to US-TENS combination and US-interferential current combination treatments. Also, HILT can be used as an effective method in combination with an appropriate exercise program including vastus medialis strengthening to reduce pain and increase functionality in the patients with PFPS.

**Supplementary Information:**

The online version contains supplementary material available at 10.1007/s10103-024-04017-y.

## Introduction

Patellofemoral pain syndrome (PFPS) is the most common pain syndrome in the knee that occurs around the patella due to overload during flexion and extension. Pain that occurs during non-traumatic activities such as squatting, running, climbing, and descending stairs is defined as anterior knee pain [1]. PFPS accounts for approximately 9–10% of all musculoskeletal complaints and 25–40% of all knee problems [2]. PFPS is a pathology with a very high incidence that causes anterior knee pain [3]. Although it has a higher incidence in the general population, especially in adolescents, young active adults, elite athletes, and military officers, it is most commonly seen in women, and its prevalence in women is 2 times higher than in men [4, 5]. In the diagnosis, subjective and objective methods such as patellofemoral compression test, pain in resistant knee extension and palpation of the patella are examined in detail. PFPS is characterized by pain in the retro patellar and peripatellar regions in the anterior part of the knee [1]. Risk factors in PFPS include weakness in functional tests, gastrocnemius, hamstring, quadriceps and iliotibial band tension, general ligament laxity, hamstring, quadriceps and hip muscle weakness, excessive quadriceps (Q) angle, and patellar compression. In addition, decreased vastus medialis obliquus (VMO) strength was associated with a significantly higher risk of PFPS, likely because it can lead to patellar instability [6, 7]. The abnormal position of patella could be caused by imbalanced muscle pull on patella, weakness of VMO, leading to excessive lateral tracking of the patella [8].

In the treatment of PFPS, conservative approaches should be preferred before invasive approaches. Strengthening the quadriceps muscle is one of them. Retraining of the VMO and general quadriceps strengthening could improve knee function and long-term pain reduction for patients with PFPS, which is the effect of decreasing force mainly in the patellofemoral joint [8]. In literature, other conservative approaches include methods such as patellar banding, strengthening hamstring, anterior tibialis, and gluteal muscles, stretching shortened structures such as iliotibial band and lateral retinaculum, modification of activities, electrophysical agents such as biofeedback, neuromuscular electrical stimulation, therapeutic ultrasound, thermotherapy, transcutaneous electrical nerve stimulation (TENS), interferential current, knee braces, and appropriate shoe selection [9, 10].

High-intensity laser therapy (HILT), which is non-invasive and painless, is currently used as a regenerative treatment in musculoskeletal diseases. Based on the time of interaction and the effective power density, the interaction between the laser beam and the tissue can be divided into three types; these are photochemical, photothermic, and photomechanical effects. High-intensity laser performs these effects thanks to its anti-inflammatory, anti-endemic, and analgesic mechanisms. Its analgesic effect is based on mechanisms of action such as slowing the transmission of pain stimulation and increasing the production of morphine-like substances [11]. Due to these effects, results of studies using HILT in patients with PFPS have shown that the high-intensity laser is more efficient in decreasing pain [12, 13].

In the literature, HILT is effective in the regenerative process of tissues, bone formation, new cartilage synthesis, and cartilage matrix synthesis [14]. It has been determined that high-intensity laser contributes to the healing process in tendon and ligament lesions and prevents the development of fibrosis [15]. Unlike other lasers, high-intensity lasers have shorter emission times and longer emission ranges, so they can reach and stimulate large and deep joints that are difficult to reach [16]. Additionally, it has participated in physical therapy applications as a modality used in many painful situations due to its photothermal, biostimulation, analgesic, and anti-inflammatory effects [17].

Although there is no consensus in the literature about the duration, pulse power, energy dose, and frequency of laser treatment in patients receiving laser therapy, a small number of studies have investigated the effects of high-intensity laser treatment on cervical radiculopathy, frozen shoulder, lateral epicondylitis, carpal tunnel syndrome, myofascial syndrome, low back pain, gonarthrosis, postmastectomy pain, and PFPS [18]. As a result, it is seen that there are few studies conducted with high-intensity laser in patients with PFPS [12, 13, 19], and no studies are comparing with different electrophysical agents. Nouri et al. investigated the efficacy of high-intensity lasers (five sessions) versus sham laser [12]. In another study, HILT (six sessions) and routine physical therapy (exercise and patellar mobilization) were compared with routine physical therapy [13]. They only examined the effect of laser on pain. In our study, HILT (ten sessions) was compared with combinations of different electrophysical agents, and patients were evaluated in more detail (VAS, Kujala, FROM, LEFS, TUG). Therefore, this study will be very useful for future researchers and clinicians as well as in filling the literature gap by revealing the importance of HILT.

We hypothesize that HILT will have a superior effect on improving pain and function in patients with PFPS than the other therapeutic methods. From this point of view, our study aims to investigate the effectiveness of high-intensity laser therapy on pain and lower extremity function by comparing it with different electrophysical agents in the treatment of PFPS.

## Materials and methods

This study is a single-blind randomized controlled study designed to investigate the effectiveness of high-intensity laser therapy on pain and lower extremity function in the treatment of PFPS. The trial was conducted in accordance with the Declaration of Helsinki and was approved by the Committee on Human Rights Related to Research involving Human Subjects, Faculty of Health Science, Istanbul Medipol University(File number:E-10840098-772.02-3259, Number: 767). The protocol of the study was registered at ClinicalTrials.gov (NCT05075525). The study was conducted in the Physiotherapy and Rehabilitation clinic of Lifemed between September 2021 and February 2023.

### Participants

It was completed with 45 volunteer individuals between the ages of 25–45 who were diagnosed with PFPS by a doctor specializing in physical medicine and rehabilitation and met the inclusion criteria. Twenty-four of the participants were women and 21 were men. Doctor performed the functional tests or queried the patient about pain with functional activities. The measures were (1) manual compression of the kneecap against the femur at rest or during an isometric knee extensor contraction, (2) palpation of the patella, (3) resisted isometric quadriceps femoris muscle contraction, (4) squatting, (5) kneeling, and (6) stair climbing. Also, magnetic resonance image was used for diagnosis. Diagnosis was made according to the ICD classification of body structures, functions, and activity and participation associated with PFPS. (ICD-10) code of PFAS is M22.2X9.

All participants were fully informed about the study, and written informed consent was obtained before treatment. Inclusion criteria were as follows: Patients diagnosed with unilateral PFPS; aged between 25 and 45 years; patients with knee pain that lasted more than 3 months, and having two or more of the symptoms that occurred without trauma such as sitting for a long time, climbing and descending stairs, running, bending the knee, jumping, and stepping down were included in the study. In addition, positive patellar compression and grind tests were also inclusion criteria. Exclusion criteria were history of previous knee pain, trauma, surgery, and other joint diseases; signs of knee osteoarthritis in knee X-ray; having neurological problems that will affect walking; pregnancy; history of chronic diseases; and the presence of malignancy and infection.

### Sample size

It was calculated using the G*power sample size calculator. To determine the sample size, a power analysis was performed to detect the effect size of score in pain improvement scale (VAS), as a primary outcome measure [20]. When the sample size was calculated with 80% power and 0.50 effect size, the number of participants to be taken was found to be 42 (*α* = 0.05, *β* = 0.50) [19]. To allow for the dropout, the sample size increased to 45 patients.

### Randomized allocation

**Fig. 1:**
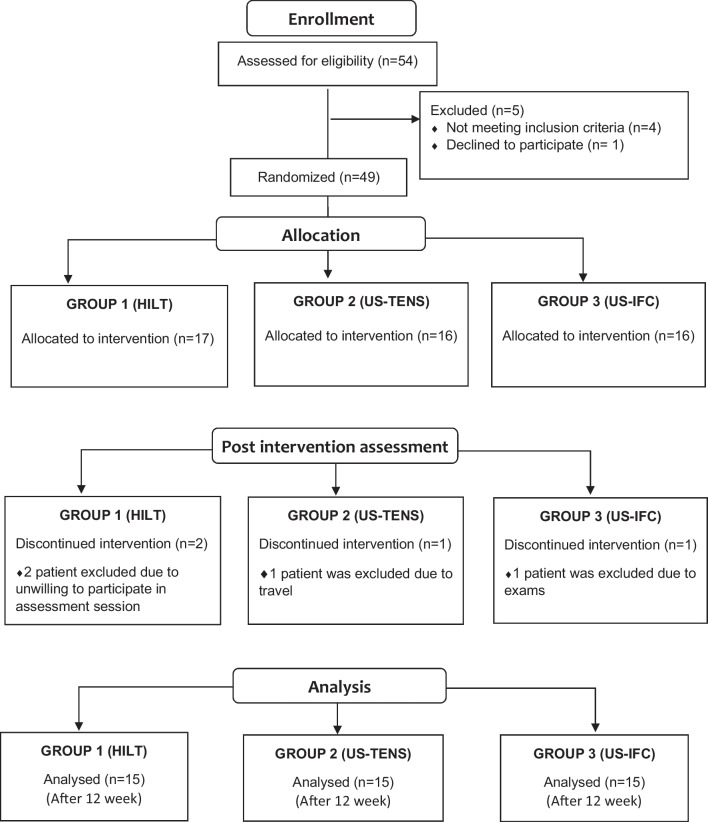
Flow diagram of the participants

Initially, 54 patients were enrolled to study. Five participants did not meet inclusion criteria. The main patients were 49 patients who were randomly divided into three groups. Four patients were excluded for different reasons at the next stage. The randomization of the groups was determined by the closed-box method. Fifteen of the numbers 1, 2, and 3 are placed in the box. A random paper was drawn for the participants included in the study. The participant with the number 1 was included in the first group, the participant with the number 2 was included in the second group, and the participant with the number 3 was included in the third group. Participants were divided into three groups: (1) HILT and exercise, (2) ultrasound-TENS and exercise, and (3) ultrasound-interferential current and exercise. Four patients were excluded due to the different reasons during the treatment period. As a result of the study, fifteen patients were included in each group (Fig. 1).

### Outcome measures

In the evaluation, the age, height, weight, and body mass index (BMI) data of all participants were recorded. Outcome measures consisted of knee pain by visual analog scale (VAS), the range of active knee flexion (FROM), Q angle, pain threshold, muscle strength measurement, Kujala patellofemoral scoring, lower extremity functional scale (LEFS), and Timed Up and Go Test (TUG).

The VAS (0–10 cm) was used to evaluate pain. The patient was asked to mark the pain severity on a 10-cm line (0: no pain, 10: pain too severe to be tolerated) [21].

Knee flexion measurement was performed with a universal goniometer in the prone position. It was measured three times for each patient, and the mean of these values was recorded in the patient evaluation form [17].

The line drawn from the anterior superior iliac spine (ASIS) to the center of the patella and the angle formed between the intersection points of the lines drawn from the tuberosity tibia to the midpoint of the patella were evaluated as the Q angle [22]. It was measured three times with a goniometer in the supine position, and the average was recorded.

Pressure pain threshold measurement was measured with an Algometer device (Baseline Push–Pull Force Gauge®, Fabrication Enterprises, Inc.). An Algometer is a device used to define pressure and pressure pain threshold [23]. At the point where they started to feel pain with the pressure force applied during the measurement, they were asked to verbally return, and the application was terminated. Three measurements were done on the medial side of the knee, the lateral side of the knee, and above the patella. Measurements were taken every 30 s, and the average of the three measurements was recorded.

A Myometer (Manual Muscle Tester, Lafayette Instruments) was used to evaluate the muscle strength of the quadriceps and hamstring. Also known as a “hand-hold dynamometer,” the Myometer is an easily portable device that allows you to measure muscle strength objectively [24]. During the muscle strength measurement, the patient was positioned appropriately. Each measurement was repeated three times, and the average of the test was recorded.

Kujala patellofemoral scoring is a scale that allows a functional evaluation of knee complaints due to the patellofemoral structure. These scoring system values are in the range of 100 (normal, painless, fully functional knee) to 0 (severe knee pain and dysfunction). The validity and reliability of the scale were conducted by Kuru et al. [25].

The LEFS was developed to evaluate the lower extremity functions, abilities, and activity limitations of individuals. It is a 20-question scale in which the lower extremity functions of the participants can be graded between 0 and 4 points, and a total of 0–80 points can be obtained. Eighty points indicate the upper limit of the functional level. The validity and reliability of the LFES were conducted by Duruturk et al. [26].

The TUG is an easy-to-apply test in which the risk of falling, walking speed, and mobility can be evaluated. It was used to evaluate lower extremity function. In the test, the patient was told to get up from the chair he was sitting in, walk to the place marked 3 m away, and return to the chair. The total elapsed time was recorded with a stopwatch. The average of the three tests was recorded. A shorter TUG test shows better functional performance. The validity and reliability of the TUG test were conducted by Yuksel et al. [27].

### Intervention

A total of ten sessions of treatment were applied to all three groups 5 days a week for 2 weeks by the same physiotherapist. Evaluations were done before, after, and 12 weeks after treatment.

**Fig. 2:**
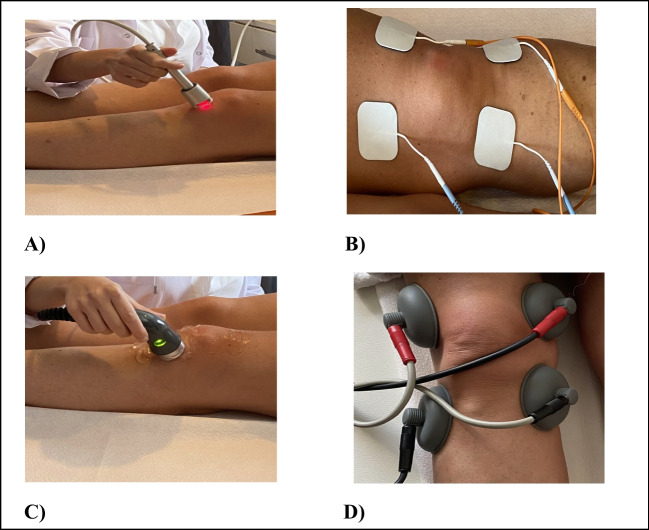
Treatment area around the patella: methods of HILT application (**A**), TENS application (**B**), US application (**C**) and interferential current application (**D**)

Group 1 (high-intensity laser): The 1064 nm wavelength Nd:YAG Laser (iLux Laser device, Mectronic Medicale) was used in the present study. The HILT application was applied to the knee area with a high-intensity laser device at a frequency of 25 Hz at a frequency of 10 watts with a dosage of 12 j/cm^2^ on an area of 25 cm^2^ for 4 min in analgesia mode and for 6 min on an area of 25 cm^2^ with a dosage of 150 j/cm^2^ with 7 watts of power in continuous mode in biostimulation mode [28]. The patients were in a supine position with the knee in extension placing the patella in its resting position. The application was made in direct contact with the skin on patellar margins with circular motions in both modes (Fig. 2).

Group 2 (ultrasound and TENS): Ultrasound was applied with an intensity of 1.5 W/cm^2^, 1 MHz, 50% intermittent for 5 min. The patients were placed in a supine position with the knee in extension, and US was applied around the patella with circular movements. TENS application was performed using four adhesive electrodes on medial and lateral parts of the knee joint at 80 Hz frequency and 50–100 ms current for 20 min. The current intensity was adjusted according to the patient’s tolerance (Fig. 2).

Group 3 (ultrasound and interferential): Ultrasound was applied with an intensity of 1.5 W/cm^2^, 1 MHz, 50% intermittent for 5 min. The patients were placed in a supine position with the knee in extension, and US was applied around the patella with circular movements. The interferential current was applied with vacuum probes at a frequency of 4 Hz, a pulse frequency of 90–100 Hz, and a 1/1 rectangular spectrum for 20 min. Four vacuum probes were placed on the medial and lateral parts of the patella (Fig. 2).

### Exercise program

All groups were given the same exercise program. The exercise program consisted of stretching and strengthening exercises, which includes weakness of the vastus medialis and hip muscles, shortening of the iliotibial tract and hamstrings, and different muscle recruitment patterns [29]. The exercises were shown to the patients by the same physiotherapist (XX) in the first session and distributed in the form of a prescription. The exercises were done in the form of 1 set of 10 repetitions, 3 sets per day during the treatment period and as a home program for 3 months after the treatment. New exercises were added to the exercises according to weeks (Table 1). Patients were asked to keep an exercise diary for the follow-up of their exercises. In addition, patients were regularly contacted by the physiotherapist to ensure their compliance with an exercise program.

**Table 1:** Exercise program

Application time	Stretching exercises	Strengthening exercises
1–2 weeks	- Hamstring stretching - Gastrocnemius stretching	- Straight leg raise - Isometric quadriceps exercise - Lateral straight leg raise - Isometric hip adduction exercise
3–8 weeks	- Hamstring stretching - Gastrocnemius stretching - Iliotibial band stretching	**In addition to the exercises of the week 1–2:** - Squat with 30° knee flexion - Bridging while holding a small ball between knees
9–14 weeks	- Hamstring stretching - Gastrocnemius stretching - Iliotibial band stretching	**In addition to the exercises of the week 3–8:** - Side lunge exercise - Forward lunge exercise

### Statistical analysis

SPSS (Statistical Package for Social Science) version 25.0 was used for statistical analysis. Descriptive statistics in the study were presented in terms of mean, standard deviation, and percentage. The Kolmogorov–Smirnov test was used to assess data normality, and it revealed all measurements had a normal distribution. Homogeneity of variances was evaluated with the Levene’s test. The ANOVA was used for comparing the mean value of demographic data at the baseline. Regardless of the homogeneity of the variances, intragroup time-dependent differences were analyzed by two-way repeated measure ANOVA to compare at the pre-post and post-treatment 3rd month. One-way ANOVA test was used to detect the significant changes in the data among the groups and in three stages of the assessments. The LSD and Bonferroni post hoc tests were also used to identify the between-group differences. The statistical significance was accepted as *p* < 0.05.

## Results

A total of 50 patients who met the inclusion criteria participated in the study, but the study was completed with 45 patients (24 females and 21 males), 15 patients in each group. The demographic data analyses of the individuals participating in the study are shown in Table 2. The mean age of group 1 was 33.06 ± 6.26, the mean age of group 2 was 34.40 ± 7.03, and the mean age of group 3 was 33.93 ± 6.80. The mean BMIs were 24.71 ± 5.14 (group 1), 26.32 ± 4.15 (group 2), and 26.68 ± 4.06 (group 3) kg/m^2^. There was no significant difference in the intergroup comparisons of demographic data. In addition, there was no statistically significant difference in clinical data between the groups in the pre-treatment evaluations (Table 3).

**Table 2:** Baseline demographic and clinical characteristics of participants

			Group 1 (*n* = 15) (mean ± SD)	Group 2 (*n* = 15) (mean ± SD)	Group 3 (*n* = 15) (mean ± SD)	*F*	*p* value
Demographics	**Age (years)**		33.06 ± 6.26	34.40 ± 7.03	33.93 ± 6.80	0.152	0.859
	**Gender (*n*/%)**	**Female**	8 (53.30)	9 (60)	7 (46.70)	0.253	0.778
		**Male**	7 (46.70)	6 (40)	8 (53.30)		
	**Height (cm)**		1.67 ± 0.08	1.65 ± 0.68	1.66 ± 0.08	0.285	0.754
	**Weight (kg)**		69.26 ± 14.02	71.93 ± 10.72	73.80 ± 12.14	0.508	0.605
	**BMI (kg/m**^**2**^**)**		24.71 ± 5.14	26.32 ± 4.15	26.68 ± 4.06	0.823	0.446

N*ote: SD* standard deviation, *BMI* body mass index

**Table 3:** Pre-treatment (week 0) and post-treatment (weeks 2 and weeks 12) comparisons of results of parameters within and between groups

		Group 1 (mean ± SD)		Group 2 (mean ± SD)	Group 3 (mean ± SD)	*F*	*p* value^a^	Group 1	Group 2	Group 3
								95% confidence interval for mean (lower and upper limits)		
FROM										
Pre-treatment		107.33 ± 12.37		111.66 ± 11.44	103.66 ± 12.02	1.684	0.198	100.481/114.185	105.329/118.003	97.009/110.324
Post-treatment		121.66 ± 9.75		115.53 ± 11.27	108.86 ± 10.35	5.597	0.007	116.262/127.071	109.289/121.777	103.135/114.598
Post-treatment 3rd month		131.66 ± 7.48		118.33 ± 9.57	113.73 ± 8.69	17.487	0.000*	127.524/135.809	113.031/123.635	108.917/118.549
*p* value^b^		0.000*		0.000*	0.000*					
Effect size		0.821		0.678	0.617					
Q angle										
Pre-treatment		14.20 ± 3.98		16.40 ± 2.66	14.13 ± 3.24	2.231	0.120	11.992//16.407	14.922/17.877	12.334/15.932
Post-treatment		14.00 ± 4.03		16.26 ± 2.60	14.06 ± 3.12	2.279	0.115	11.765/16.234	14.824/17.708	12.334/15.798
Post-treatment 3rd month		12.53 ± 3.66		15.33 ± 2.28	13.00 ± 3.00	3.663	0.034	10.505/14.561	14.065/16.600	11.338/14.661
*p* value^b^		0.000*		0.001*	0.000*					
Effect size		0.810		0.490	0.700					
Muscle strength	Hamstring									
	Pre-treatment Post-treatment Post-treatment 3rd month *p* value^b^ Effect size		3.49 ± 0.80 4.67 ± 1.01 5.38 ± 1.02 0.000* 0.827	3.99 ± 0.73 4.26 ± 0.58 4.49 ± 0.64 0.000* 0.658	3.24 ± 0.90 3.47 ± 0.68 3.87 ± 0.64 0.003* 0.400	3.214 9.092 13.747	0.050 0.001* 0.000*	3.046/3.937 4.112/5.239 4.812/5.948	3.583/4.397 3.937/4.582 4.137/4.847	2.746/3.749 3.093/3.853 3.517/4.229
	Quadriceps									
	Pre-treatment Post-treatment Post-treatment 3rd month *p* value^b^ Effect size		3.30 ± 0.68 4.11 ± 0.72 5.00 ± 0.70 0.000* 0.861	3.92 ± 0.91 4.02 ± 0.85 4.27 ± 0.74 0.017 0.300	3.30 ± 0.55 3.57 ± 0.51 3.75 ± 0.53 0.001* 0.480	3.558 2.478 13.423	0.037 0.096 0.000*	2.923/3.683 3.713/4.516 4.614/5.400	3.418/4.426 3.557/4.501 3.861/4.684	2.993/3.614 3.293/3.860 3.457/4.044
Pain threshold	Upper side of patella									
	Pre-treatment Post-treatment Post-treatment 3rd month *p* value^b^ Effect size		2.76 ± 0.30 3.44 ± 0.28 3.70 ± 0.07 0.000* 0.852	2.94 ± 0.53 3.22 ± 0.38 3.36 ± 0.48 0.027 0.233	2.83 ± 0.51 3.12 ± 0.42 3.11 ± 0.41 0.007 0.311	0.579 2.814 9.762	0.565 0.071 0.000*	2.597/2.936 3.283/3.596 3.662/3.750	2.649/3.244 3.014/3.438 3.100/3.632	2.550/3.116 2.889/3.363 2.884/3.342
	Medial side of knee									
	Pre-treatment Post-treatment Post-treatment 3rd month *p* value^b^ Effect size		2.46 ± 0.37 3.17 ± 0.40 3.63 ± 0.25 0.000* 0.825	2.69 ± 0.74 3.21 ± 0.84 3.02 ± 0.74 0.088 0.163	2.56 ± 0.52 2.72 ± 0.40 2.94 ± 0.41 0.019 0.271	0.638 3.264 8.103	0.534 0.048 0.001*	2.250/2.669 2.950/3.396 3.490/3.776	2.282/3.104 2.745/3.680 2.609/3.430	2.270/2.284 2.498/2.941 2.715/3.177
	Lateral side of knee									
	Pre-treatment Post-treatment Post-treatment 3rd month *p* value^b^ Effect size		2.50 ± 0.52 3.24 ± 0.49 3.72 ± 0.08 0.000* 0.766	2.80 ± 0.70 3.07 ± 0.67 3.18 ± 0.66 0.035 0.237	2.72 ± 0.52 3.01 ± 0.44 3.26 ± 0.45 0.003* 0.344	1.047 0.733 5.712	0.360 0.487 0.006	2.218/2.794 2.969/3.523 3.672/3.767	2.416/3.196 2.700/3.446 2.818/3.555	2.438/3.014 2.766/3.260 3.007/3.512
VAS										
Pre-treatment		6.80 ± 1.74		6.86 ± 2.09	6.46 ± 1.76	0.196	0.823	5.836/7.763	5.703/8.029	5.487/7.445
Post-treatment		0.93 ± 1.38		4.73 ± 2.96	3.20 ± 2.21	10.549	0.000*	0.165/1.701	3.092/6.374	1.975/4.424
Post-treatment 3rd month		0.00 ± 0.00		3.26 ± 2.05	2.26 ± 1.66	18.034	0.000*	0.000/0.000	2.130/4.402	1.343/3.190
*p* value^b^		0.000*		0.000*	0.000*					
Effect size		0.922		0.719	0.757					
Kujala										
Pre-treatment		64.40 ± 14.53		66.40 ± 10.08	64.40 ± 13.17	0.123	0.884	56.351/72.449	60.815/71.984	57.104/71.695
Post-treatment		89.86 ± 7.55		74.46 ± 10.39	76.06 ± 10.15	12.018	0.000*	85.681/94.052	68.708/80.225	70.440/81.692
Post-treatment 3rd month		90.40 ± 22.42		78.53 ± 11.83	79.60 ± 6.84	2.812	0.071	77.980/102.819	71.981/85.085	75.810/83.389
*p* value^b^		0.002*		0.000*	0.000*					
Effect size		0.469		0.536	0.629					
LEFS										
Pre-treatment		50.73 ± 14.96		49.26 ± 14.37	49.26 ± 14.65	0.050	0.951	42.446/59.020	41.306/57.227	41.150/57.382
Post-treatment		74.40 ± 8.50		57.13 ± 14.20	59.00 ± 12.22	9.535	0.000*	69.688/79.112	49.265/65.000	52.227/65.772
Post-treatment 3rd month		77.66 ± 3.06		63.26 ± 13.00	62.80 ± 10.11	11.449	0.000*	75.970/79.362	56.066/70.467	57.198/68.401
*p* value^b^		0.000*		0.000*	0.000*					
Effect size		0.742		0.599	0.675					
TUG										
Pre-treatment		8.19 ± 2.00		8.53 ± 1.43	9.27 ± 2.50	1.116	0.337	7.079/9.304	7.741/9.329	7.888/10.662
Post-treatment		6.76 ± 1.37		8.11 ± 1.28	8.36 ± 1.97	4.467	0.017	6.006/7.531	7.404/8.831	7.274/9.457
Post-treatment 3rd month		5.78 ± 0.80		7.52 ± 1.16	7.73 ± 1.50	12.022	0.000*	5.339/6.232	6.884/8.173	6.895/8.568
*p* value^b^		0.000*		0.000*	0.000*					
Effect size		0.690		0.651	0.563					

*FROM* flexion range of motion, *VAS* visual analog scale, *LEFS* lower extremity functional scale, *TUG* Timed Up and Go Test

*Significant *p* values (*p* < 0.05)

^a^*p* value for measurements among the three groups (ANOVA test)

^b^*p* value comparison of within-group measurements for each group (repeated measure ANOVA test)

### Intragroup comparisons

Intragroup comparison of the measurements of the groups is shown in Table 3. A statistically significant difference was found in all intragroup evaluation measurements of the 1st group (*p* < 0.05). When the intragroup evaluation measurements of the 2nd group were examined, no statistically significant difference was found in the measurements of quadriceps muscle strength (95% confidence interval (CI), 3.861/4.684; *p* = 0.017) and pain threshold values (*p* > 0.05). A statistically significant difference was found in all other evaluation measurements. When the intragroup evaluation measurements of the 3rd group were examined, no statistically significant difference was found in the pressure pain threshold value measured from above the patella (95% CI, 2.884/3.342; *p* = 0.007) and medial side of the knee (95% CI, 2.715/3.177; *p* = 0.019). A statistically significant difference was found in all other evaluation measurements (*p* < 0.05).

### Comparisons between groups

The comparison of the measurements of the groups before, after, and at the 3rd month after treatment is shown in Table 3.

As a result of the post-treatment measurements, a statistically significant difference was found between the groups in hamstring muscle strength (95% CI, 3.860/4.412), VAS (95% CI, 2.135/3.775), Kujala patellofemoral score (95% CI, 76.651/83.615), and LEFS (95% CI, 59.304/67.718) values (*p* < 0.05; Table 3). There was no statistically significant difference between the groups in knee flexion angle (95% CI, 111.893/118.817), Q angle (95% CI, 13.775/15.800), quadriceps muscle strength (95% CI, 3.686/4.127), pressure pain thresholds, and TUG (95% CI, 7.242/8.260) test values (*p* > 0.05; Table 3).

In the measurements performed during the 3rd month after treatment, a statistically significant difference was found between the groups in knee flexion angle (95% CI, 117.816/127.672), hamstring (95% CI, 4.283/4.880) and quadriceps muscle strength (95% CI, 4.092/4.594), pressure pain threshold value measured from the medial of the knee (95% CI, 3.022/3.377) and above the patella (95% CI, 3.264/3.526), VAS (95% CI, 1.233/2.455), LEFS (95% CI, 64.381/71.440), and TUG (95% CI, 6.575/7.455) test values (*p* < 0.05; Table 3). There was no statistically significant difference in the Q angle (95% CI, 12.656/14.587), pain threshold value measured from the lateral side of the knee (95% CI, 3.233/3.544), and Kujala patellofemoral scores (95% CI, 78.105/87.583) between the groups (*p* > 0.05; Table 3).

The change of the parameters according to the time (pre-treatment, post-treatment, and post treatment 3rd month) between the groups was shown in Supplementary Fig. 1 and 2. Considering the change of all measured data between the groups over time, it was found that the effect size was greatest in all outcome measurements in group 1 (*p* < 0.05; Supplementary Fig. 1 and 2). A comparison of groups in pairs was given as Supplementary Table 1–3.

## Discussion

In our study examining the HILT effect in patients with PFPS, HILT was compared with different electrophysical agents. As a result of the study, HILT was superior to other groups at the end of 12 weeks after treatment in increasing knee flexion angle, decreasing pain, increasing Kujala patellofemoral scores, and improving lower extremity function in patients with PFPS.

There are studies in the literature describing the positive effects of high-intensity laser treatment on musculoskeletal disorders [30]. However, there are few studies examining its effectiveness on PFPS. In addition, this study was conducted because there was no study examining the long-term effectiveness of high-intensity laser therapy in PFPS by comparing it with combinations of different electrophysical agents.

PFPS is more prevalent in the general population, especially in adolescents and young active adults, but it occurs most frequently in women. Its prevalence is between 15 and 45% [4]. The age ranges of the patients participating in our study were in the range of 25–45 years, as in the results of the research, and it was observed that women were more than men. Although PFPS affects 85% of all age groups, it can recur in up to 90% of patients. Due to its high prevalence and complaints that last for an average of 20 years, it creates a huge economic burden on medical expenditures for countries [31]. Therefore, we aimed to investigate the effectiveness of high-intensity laser therapy on pain and lower extremity function in the treatment of PFPS.

### Flexion range of motion

Steinberg et al. reported that the knee flexion angles of dancers with PFPS decreased compared to those without a diagnosis of PFPS [32]. In our study, the decreased knee flexion angles of patients with PFPS increased as a result of treatment. Nazari et al. compared HILT, exercise therapy, and conventional therapy in patients with knee osteoarthritis and found HILT to be effective in increasing knee flexion angle [20]. Akaltun et al. [33] compared HILT with a placebo laser in their study examining the effectiveness of HILT in patients with knee osteoarthritis and stated that HILT was more effective than the other group in increasing knee flexion angle. Similarly, Alayat et al. stated that HILT and exercise increased the lumbar range of motion [34]. Venosa et al. compared HILT and US-TENS combination therapy in patients with cervical spondylosis and found HILT to be more effective in increasing the cervical range of motion [35]. Nouri et al. examined the efficacy of HILT on pain and function in PFPS but did not evaluate the knee flexion angle [12].

According to the literature, it is seen that HILT increases ROM when applied in different regions. As a result of our study, an increase in flexion angle was found in all groups. There was a higher increase in knee flexion angle in the HILT group. We think that this is because HILT provides an increase in flexibility in deep tissues with the effect of heating and has a high pain reduction effect.

### Q angle

As a result of our study, there was no difference in the Q angle at the end of the treatment. However, in the evaluation at the 3rd month after the treatment, there was a statistically significant improvement in the Q angle in all groups, and the superiority of the groups was not found.

The normal Q angle is 8–12° in males and 15–18° in females. Generally, a Q angle greater than 20° is considered abnormal. In this study, there was no patient with a Q angle above 20°. Although the Q angle is frequently associated with PFPS in the literature, there is no consensus on the functional importance of the Q angle [36].

Lee et al., in their study on elite athletes with PFPS, found that therapeutic exercise performed 3 days a week for 8 weeks reduced the Q angle [37]. Tunay et al. [38] divided patients with PFPS into four groups and applied different treatment approaches for 3 weeks, and as in our study, there was a decrease in Q angle after treatment in all treatment groups. As a result of the treatment with exercise, there is a decrease in the Q angle with an increase in muscle strength and the balance of the vastus medialis obliquus/vastus lateralis muscle. Therefore, we believe that exercise and HILT should not be neglected in patients with PFPS. In our study, the decrease in Q angle was greater in the laser group. We consider that this is due to the improvement in muscle strength of the knee.

### Muscle strength

According to our results, there was a statistically significant increase in hamstring muscle strength was found in all groups. A significant improvement was found in quadriceps muscle strength in all groups except the US-TENS group. The highest increase in muscle strength among the groups was observed in the HILT and exercise groups. Yılmaz et al. administered HILT to patients with subacromial impingement syndrome and found that the HILT group was more effective in increasing muscle strength than the placebo group [39]. Karaca et al. applied HILT and extracorporeal shock wave therapy to patients with lateral epicondylitis. The highest increase in grip strength was found in the HILT group [40].

Shimoura et al. examined the efficacy of TENS on pain and physical performance in patients with knee pain. As a result of their study, although they found TENS to be effective in increasing pain and walking distance, they did not find a significant increase in extensor muscle strength [41]. Alqualo-Costa et al. evaluated extensor muscle strength in their study examining the effect of interferential current and low-density laser in patients with knee osteoarthritis (OA). They stated that there was a greater increase in muscle strength in the group in which interferential current and laser were applied together than in the group in which only interferential current was applied [42].

According to the literature, no studies are looking at the short- and long-term effects of HILT on the increase in muscle strength in PFPS. As a result of our study, it was concluded that HILT can be used as an effective method in the treatment of PFPS to increase muscle strength and ROM. Thus, by strengthening the vastus medialis muscle improved knee function and reduced pain in patients with PFPS. However, there is still a need for further studies in which different doses of HILT are administered in PFPS, and different evaluations are done.

### Pain threshold

As a result of our study, there was no statistically significant difference in pain threshold values between the groups after the treatment. A statistically significant increase was found in the HILT and US-interferential current groups in the evaluation performed after 3 months. The highest increase was observed in the HILT group. In contrast to our study, Naruseviciute et al. [43] reported that they did not find any significant difference between the groups in terms of pain threshold in their study comparing the effectiveness of low- and high-intensity lasers. Aceituno-Gómez et al. [44] compared HILT with sham laser in patients with shoulder impingement syndrome and found no significant improvement in pain threshold despite a significant decrease in pain as a result of their study. Similar to these studies, Alqualo-Costa et al. found no significant difference in pain threshold parameter in the groups in their study comparing the combinations of interferential current and laser in people with knee pain [42]. This may be since the laser protocols and regions applied in the studies are different.

#### VAS

According to our findings, there was a significant decrease in VAS values in all groups. The greatest decrease was observed in the HILT group. Nouri et al. also found HILT to be effective in reducing pain in patients with PFPS [12]. Similar to these studies, Siriratna et al. [45], Akaltun et al. [33], and Štiglić-Rogoznica et al. [46] found HILT to be effective in reducing pain in patients with knee osteoarthritis.

The analgesic and anti-inflammatory activity of the laser is explained by many mechanisms. With the detection of pain in the sensory nerve endings, laser reduces the spasm in the muscle arterioles and creates reactive vasodilation. It has an analgesic and anti-inflammatory effect by increasing regeneration and beta-endorphins in the rheumatoid synovial membrane with protein synthesis [17]. This mechanism supports the effectiveness of HILT on pain, as in the result of our study.

Similar to the literature, Venosa et al. [35] compared US-TENS combination therapy and HILT to find both methods effective in reducing neck pain but HILT was more effective. Adedoyin et al. [47] examined the effect of TENS and interferential current on pain and functionality in patients with knee osteoarthritis. No improvement was found in both groups. In our study, we observed similar results in TENS and interferential current groups in reducing pain in patients with PFPS.

Kim et al. [48], on the other hand, found HILT to be more effective in reducing pain as a result of 4 weeks of treatment in their study in which they divided patients with knee osteoarthritis into two groups as HILT group and conservative treatment (interferential current and US).

Unlike these studies, we compared three different treatment combinations in our study. As a result of our study, it is seen that HILT is a more effective method in reducing pain in PFPS in the long term as it provides ease of use due to its short application time compared to other conventional treatment modalities.

### Kujala

A statistically significant difference was found between the groups in Kujala patellofemoral scores after the treatment. In the evaluation at the 3rd month after the treatment, no statistically significant difference was found between the groups. Nouri et al. [12] compared HILT (2-min pain relief program, 5 sessions) with the sham group and showed that there was no difference between the groups in Kujala scores. In our study, unlike this study, the highest increase in Kujala scores was observed in the HILT group (10-min HILT, 10 sessions). Quadriceps weakness, tightness of hamstring, iliotibial band, and gastrosoleus muscles are among the risk factors for PFPS. The lack of functional results of their study may be due to the lack of an exercise protocol includes weakness of the vastus medialis and hip muscles, shortening of the muscles. Also, we think that this situation depends on the different laser durations and treatment durations. New studies are needed due to the limited number of studies examining the effectiveness of HILT in the treatment of PFPS.

### Functional tests

In the data obtained from the LEFS and TUG test, a significant improvement was observed in all groups at the 3rd month after treatment. The highest functional increase was observed in the HILT group. According to the literature, Viliani et al. [49] also stated that there was a statistically significant difference in the functional evaluations of patients with knee pain in the HILT group. Nazari et al. [20] compared HILT with conventional treatment (US-TENS-Exercise) in patients with knee osteoarthritis. They found that the HILT and exercise therapy group were superior in improving functionality. Similar to this study, Kim et al. [48] compared HILT with the US and interferential current therapy as a conservative treatment and stated that HILT was more effective in increasing the function of patients with knee pain.

Samaan et al. [50] compared HILT and low-intensity pulsed ultrasound treatment with exercise in patients with knee osteoarthritis. As a result of the study, they obtained better results in knee ROM, VAS, and functionality in the HILT group. This can be explained by the analgesic effect of the laser. Laser therapy changes the release of bradykinin and histamine from damaged tissue and increases the pain threshold by increasing substance P release from peripheral nociception. In addition, HILT also increases lymphatic drainage and reduces swelling [51]. The superiority of HILT in pain and function in PFPS compared to other groups may be due to these reasons. In addition, as our study results show, we think that there is an increase in the functionality of patients with decreased pain, increased knee flexion, angle and increased muscle strength.

In the literature, HILT is a current treatment approach, and there are few studies examining the efficacy of HILT in PFPS. Additionally, there are differences in the HILT protocols and treatment durations applied in the studies [12, 13]. Our study is the first to compare HILT with combinations of different electrophysical agents in the treatment of PFPS. Therefore, our study will contribute to the literature.

Ultrasound-TENS combination and ultrasound-IFC combination methods were also found to be effective in pain and functionality. However, HILT was found to be more effective than these physical agents. These combinations can be used as an alternative method in clinics where HILT is not available.

The limitation of our study is that sonographic evaluation was not used to for following repair progress. It could be used to increase reliability. Additionally, the therapist was not blind to the groups, which may have caused bias in the results. Therefore, future studies in the future are necessary to examine the efficacy of HILT in the treatment of PFPS by using different protocols on an increased population of patients. Moreover, only the exercise group can be added to the study to better examine the effect of the HILT.

## Conclusion

According to the results of this study, high-intensity laser therapy (HILT) was found to be a more effective method in the treatment of PFPS after 3 months of follow-up compared to US-TENS combination and US-interferential current combination treatments. In summary, HILT can be used as an effective method in combination with an appropriate exercise program including vastus medialis strengthening to reduce pain and increase functionality in the patients with PFPS. Instead of using more than one physical agent in the treatment of PFPS, an effective result can be achieved in the long term with a single effective method such as laser.

## Supplementary Information

Below is the link to the electronic supplementary material.

Supplementary file1 (DOCX 1523 KB)
