# Supplementary material for: The effect of high-intensity laser therapy on pain and lower extremity function in patellofemoral pain syndrome: a single-blind randomized controlled trial
Source: Lasers Med Sci. 2024 Apr 17;39(1):103. doi: 10.1007/s10103-024-04017-y (PMC11024020; doi:10.1007/s10103-024-04017-y)
Supplement: Supplementary file 1 — Supplementary file1 (DOCX 1523 KB) [file 10103_2024_4017_MOESM1_ESM.docx]

(A) (B)
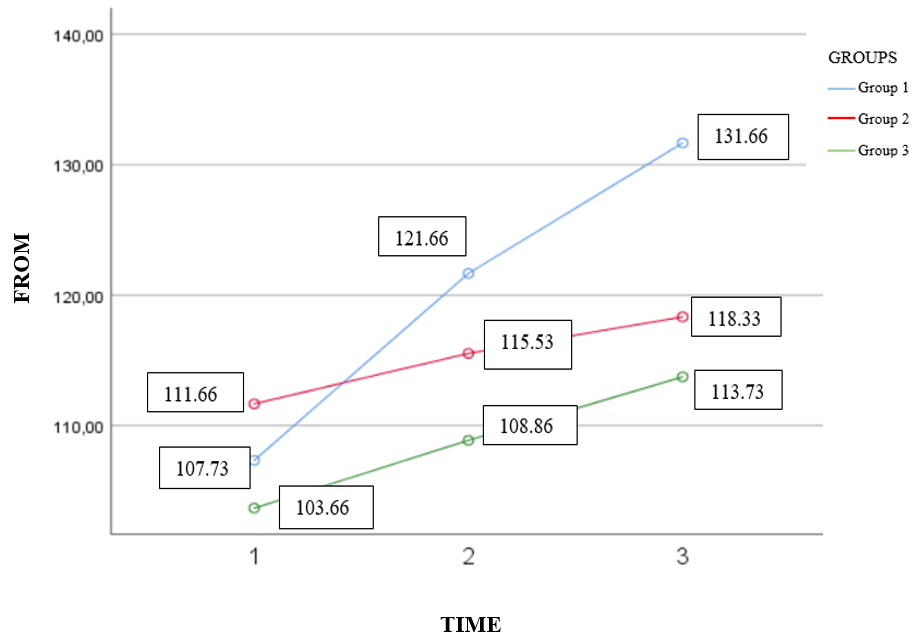

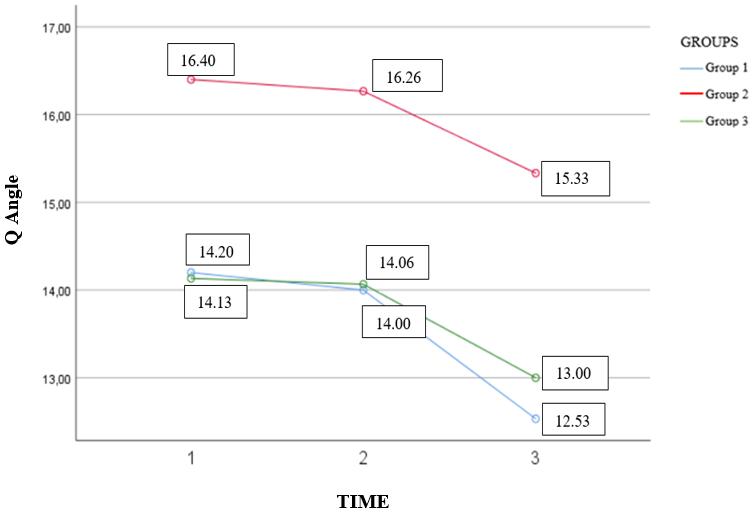


(C) (D)


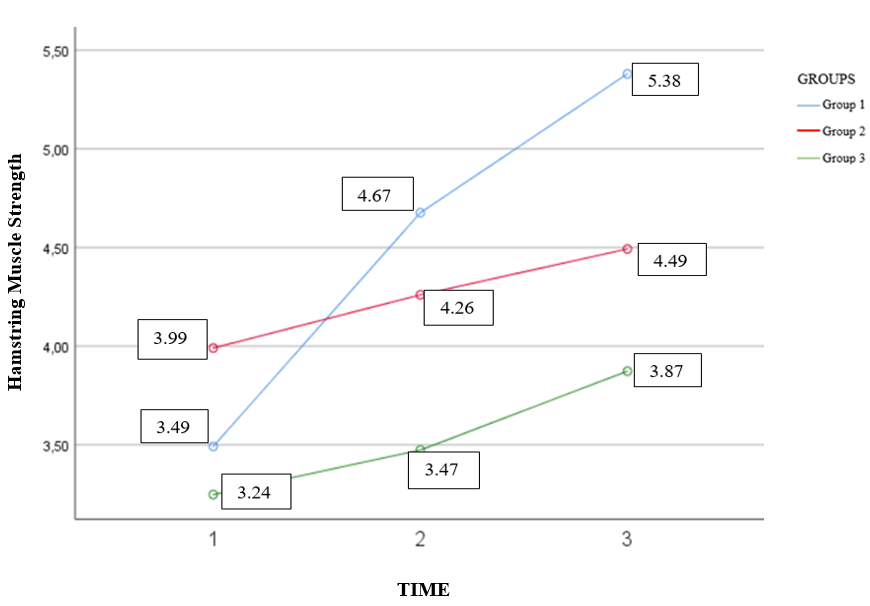

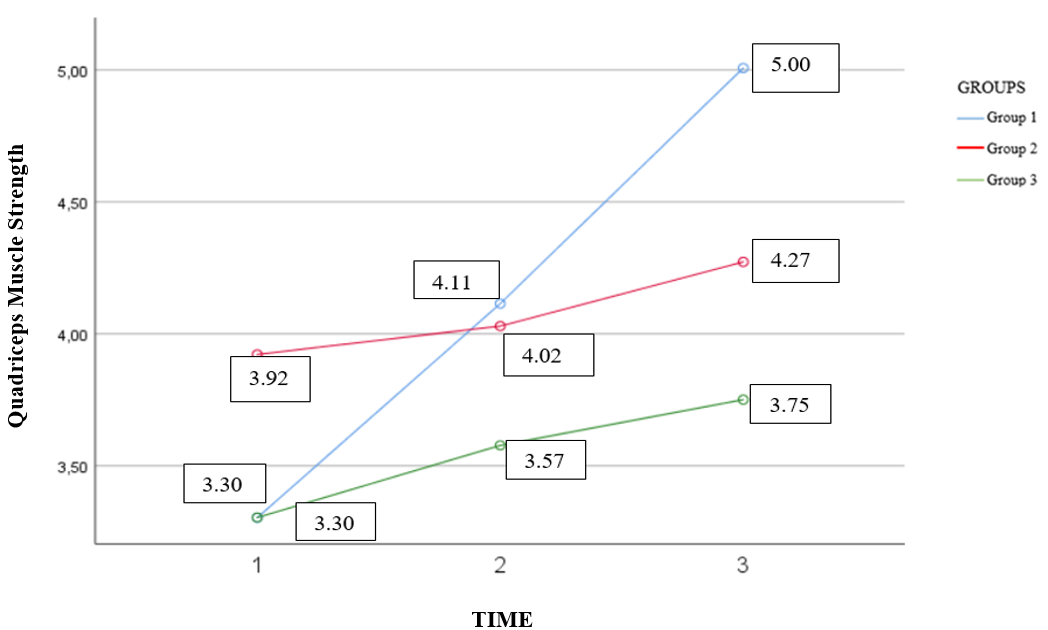


**Supplementary Fig. 1 (A)** FROM, **(B)** Q Angle,**(C)** Hamstring Muscle Strength, **(D)** Quadriceps Muscle Strength changes among groups over time

1. (B)


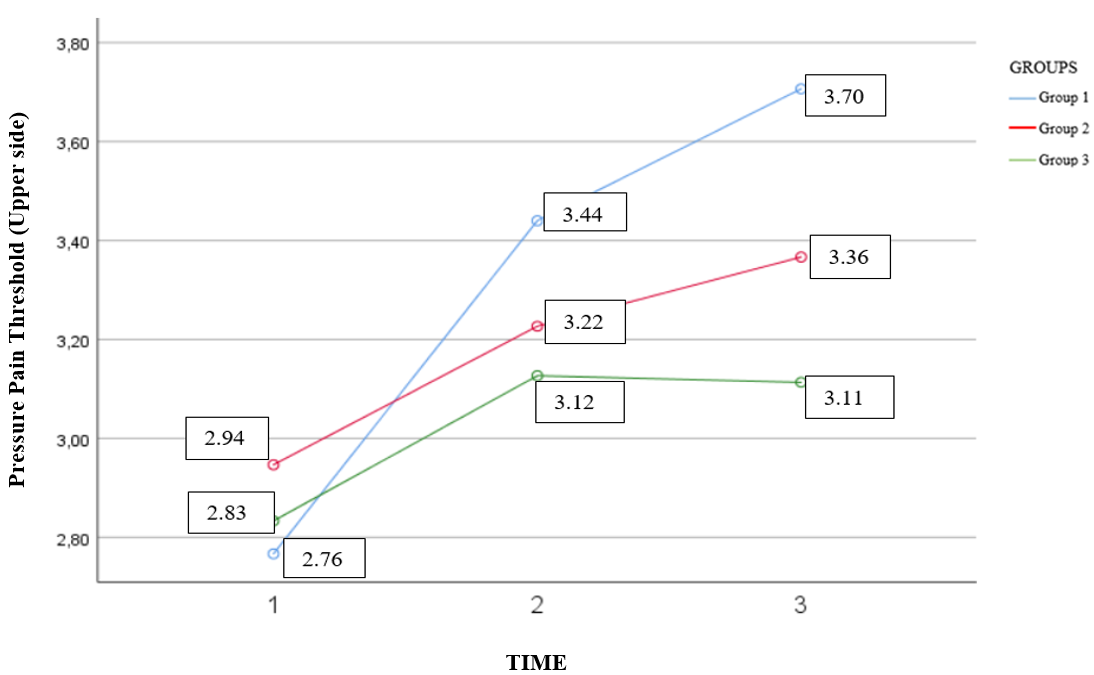

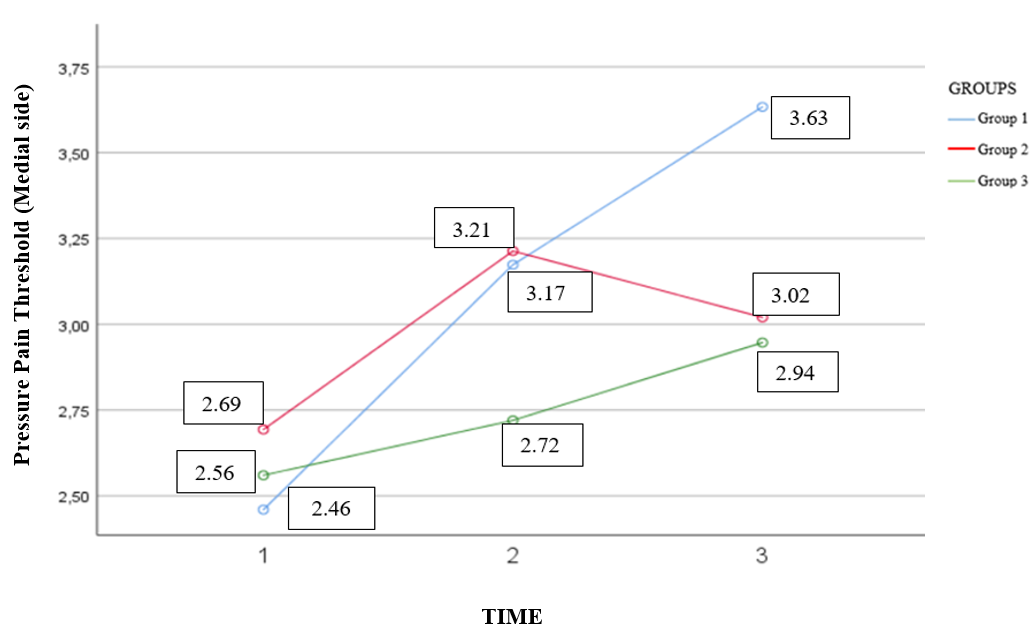


(C) (D)


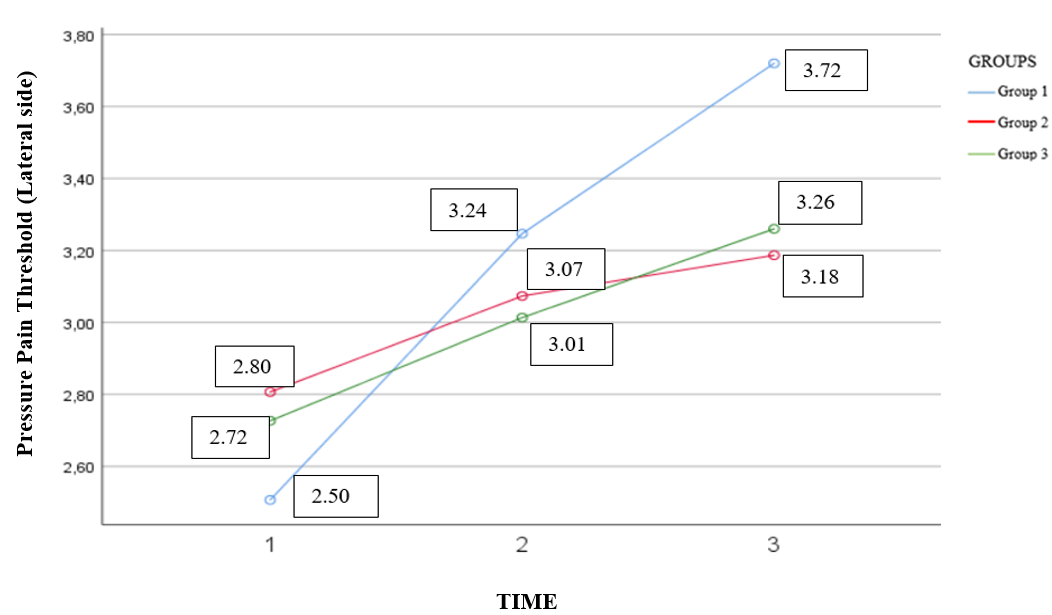

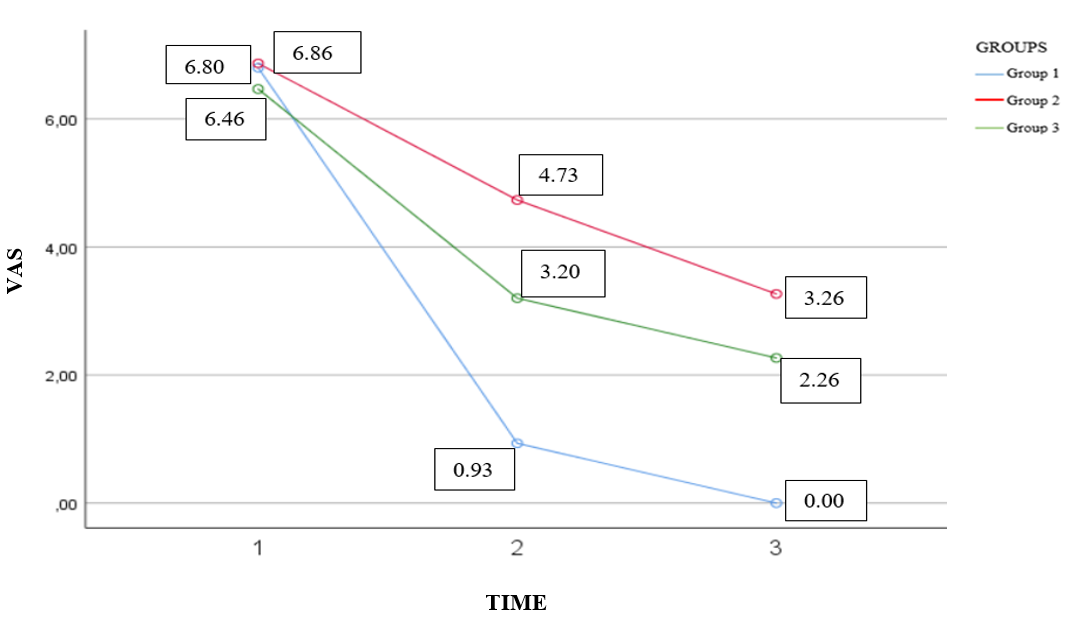


(E) (F)


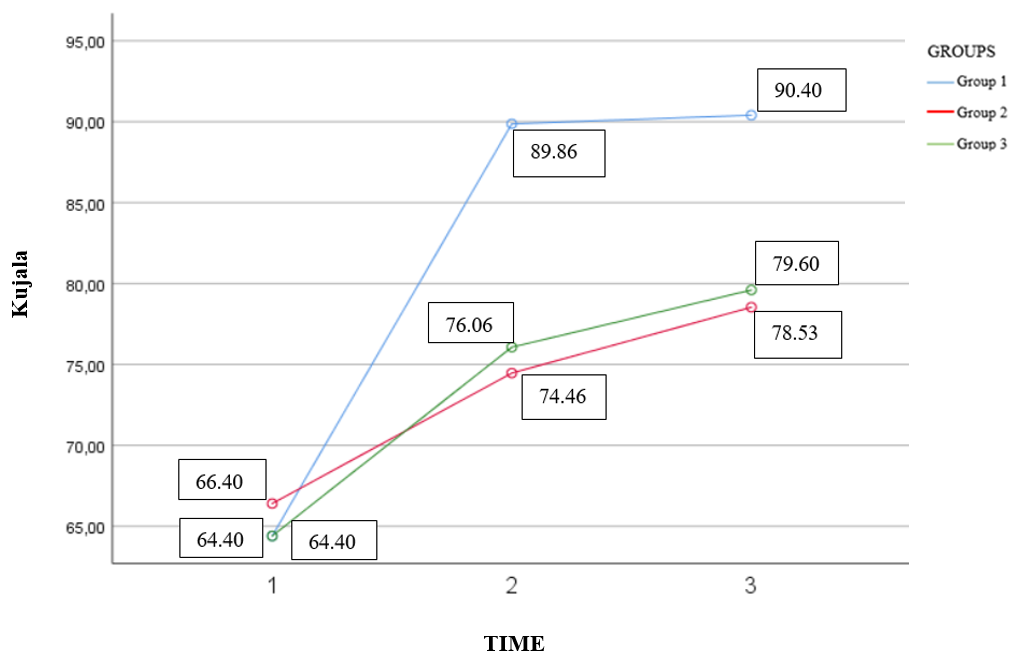

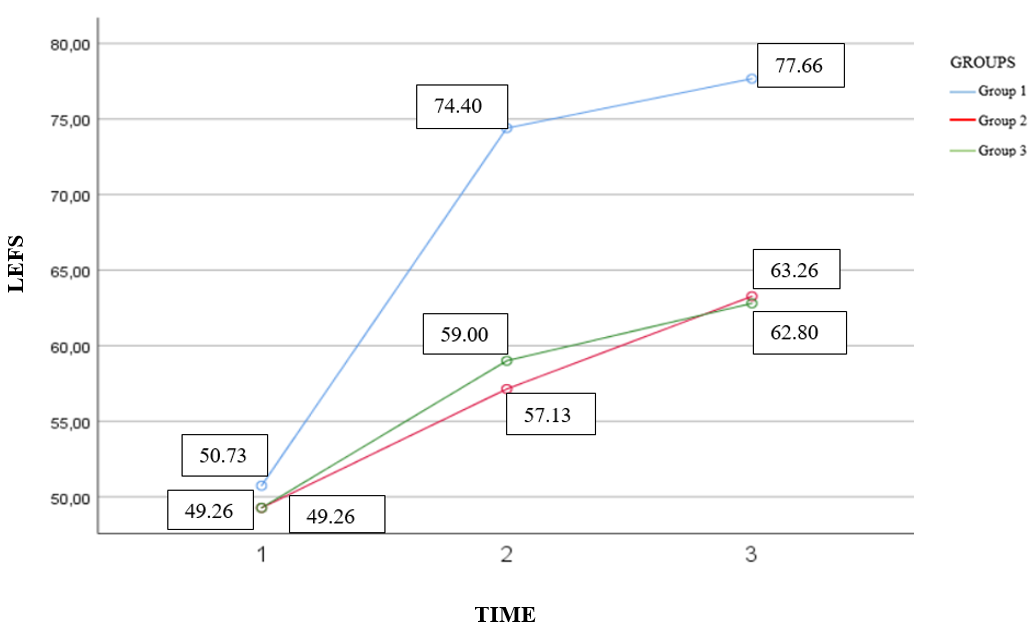


(G)


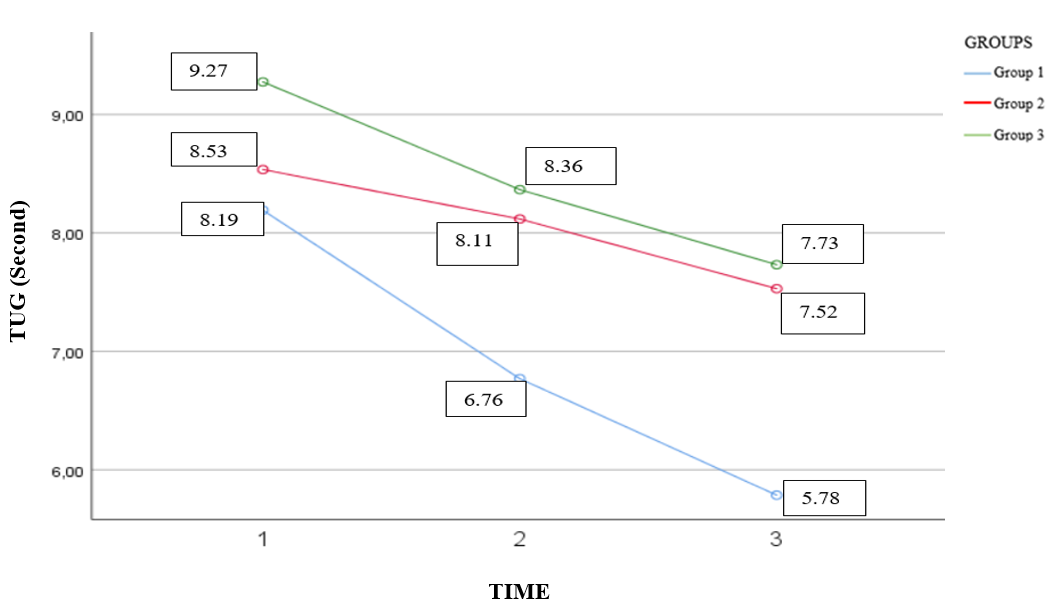


**Supplementary Fig. 2 (A)** Pressure Pain Threshold (Upper side), **(B)** Pressure Pain Threshold (Medial side)**, (C)** Pressure Pain Threshold (Lateral side)**, (D)** VAS, **(E)** Kujala Score, **(F)** Lower Extremity Functional Scale (LEFS), **(G)** TUG scores changes among groups over time

**Supplementary Table 1** Post-treatment (weeks 2 and weeks 12) comparisons of results of parameters between groups

|  | | **Mean difference** | **95% CI**  **(upper and lower limits)** | **p value** |
| --- | --- | --- | --- | --- |
| **FROM**  **Post-treatment** | **Group 1 vs Group 2** | 6.133 | 15.675/3.409 | 0.349 |
|  | **Group 1 vs Group 3** | 12.800 | 22.342/3.257 | 0.005**^*^** |
|  | **Group 2 vs Group 3** | 6.666 | 16.209/2.875 | 0.266 |
| **FROM**  **Post-treatment 3rd month** | **Group 1 vs Group 2** | 13.333 | 21.188/5.478 | 0.000**^*^** |
|  | **Group 1 vs Group 3** | 17.933 | 25.788/10.078 | 0.000**^*^** |
|  | **Group 2 vs Group 3** | 4.600 | 12.455/3.255 | 0.455 |
| **Q Angle**  **Post-treatment** | **Group 1 vs Group 2** | 2.266 | 0.746/5.279 | 0.203 |
|  | **Group 1 vs Group 3** | 0.066 | 2.946/3.079 | 1.000 |
|  | **Group 2 vs Group 3** | 2.200 | 5.213/0.813 | 0.227 |
| **Q Angle**  **Post-treatment 3rd month** | **Group 1 vs Group 2** | 2.800 | 0.035/5.564 | 0.046 |
|  | **Group 1 vs Group 3** | 0.466 | 2.297/3.230 | 1.000 |
|  | **Group 2 vs Group 3** | 2.333 | 5.097/0.430 | 0.124 |
| **Muscle Strength Hamstring**  **Post-treatment** | **Group 1 vs Group 2** | 0.416 | 1.130/0.298 | 0.462 |
|  | **Group 1 vs Group 3** | 1.202 | 1.917/0.488 | 0.000^*^ |
|  | **Group 2 vs Group 3** | 0.786 | 1.501/0.072 | 0.027 |
| **Muscle Strength Hamstring**  **Post-treatment 3rd month** | **Group 1 vs Group 2** | 0.887 | 1.607/0.167 | 0.011 |
|  | **Group 1 vs Group 3** | 1.506 | 2.227/0.786 | 0.000^*^ |
|  | **Group 2 vs Group 3** | 0.619 | 1.339/0.101 | 0.114 |
| **Muscle Strength Quadriceps**  **Post-treatment** | **Group 1 vs Group 2** | 0.085 | 0.732/0.561 | 1.000 |
|  | **Group 1 vs Group 3** | 0.537 | 1.184/0.109 | 0.134 |
|  | **Group 2 vs Group 3** | 0.452 | 1.099/0.195 | 0.266 |
| **Muscle Strength Quadriceps**  **Post-treatment 3rd month** | **Group 1 vs Group 2** | 0.734 | 1.342/0.127 | 0.013 |
|  | **Group 1 vs Group 3** | 1.256 | 1.864/0.649 | 0.000^*^ |
|  | **Group 2 vs Group 3** | 0.522 | 1.129/0.085 | 0.114 |

FROM, flexion range of motion; CI, confidence interval

^*^ Significant p values (P<0.05)

ANOVA Post-hoc test

**Supplementary Table 2** Post-treatment (weeks 2 and weeks 12) comparisons of results of parameters between groups

|  | | **Mean difference** | **95% CI**  **(upper and lower limits)** | **p value** |
| --- | --- | --- | --- | --- |
| **Pain Threshold**  **Upper side of patella**  **Post-treatment** | **Group 1 vs Group 2** | 0.213 | 0.549/0.123 | 0.364 |
|  | **Group 1 vs Group 3** | 0.313 | 0.649/0.023 | 0.075 |
|  | **Group 2 vs Group 3** | 0.100 | 0.436/0.236 | 1.000 |
| **Pain Threshold**  **Upper side of patella**  **Post-treatment 3rd month** | **Group 1 vs Group 2** | 0.340 | 0.676/0.004 | 0.047 |
|  | **Group 1 vs Group 3** | 0.593 | 0.929/0.257 | 0.000**^*^** |
|  | **Group 2 vs Group 3** | 0.253 | 0.589/0.082 | 0.201 |
| **Pain Threshold**  **Medial side of knee**  **Post-treatment** | **Group 1 vs Group 2** | 0.040 | 0.494/0.574 | 1.000 |
|  | **Group 1 vs Group 3** | 0.453 | 0.988/0.081 | 0.122 |
|  | **Group 2 vs Group 3** | 0.493 | 1.028/0.041 | 0.079 |
| **Pain Threshold**  **Medial side of knee**  **Post-treatment 3rd month** | **Group 1 vs Group 2** | 0.613 | 1.080/0.146 | 0.006 |
|  | **Group 1 vs Group 3** | 0.686 | 1.153/0.219 | 0.002**^*^** |
|  | **Group 2 vs Group 3** | 0.073 | 0.540/0.393 | 1.000 |
| **Pain Threshold**  **Lateral side of knee**  **Post-treatment** | **Group 1 vs Group 2** | 0.173 | 0.672/0.325 | 1.000 |
|  | **Group 1 vs Group 3** | 0.233 | 0.732/0.265 | 0.751 |
|  | **Group 2 vs Group 3** | 0.060 | 0.559/0.439 | 1.000 |
| **Pain Threshold**  **Lateral side of knee**  **Post-treatment 3rd month** | **Group 1 vs Group 2** | 0.533 | 0.959/0.106 | 0.010 |
|  | **Group 1 vs Group 3** | 0.460 | 0.886/0.033 | 0.031 |
|  | **Group 2 vs Group 3** | 0.073 | 0.353/0.499 | 1.000 |
| **VAS**  **Post-treatment** | **Group 1 vs Group 2** | 3.800 | 1.724/5.875 | 0.000**^*^** |
|  | **Group 1 vs Group 3** | 2.266 | 0.190/4.342 | 0.028 |
|  | **Group 2 vs Group 3** | 1.533 | 3.609/0.542 | 0.218 |
| **VAS**  **Post-treatment 3rd month** | **Group 1 vs Group 2** | 3.266 | 1.876/4.656 | 0.000**^*^** |
|  | **Group 1 vs Group 3** | 2.266 | 0.876/3.656 | 0.001**^*^** |
|  | **Group 2 vs Group 3** | 1.000 | 2.390/0.390 | 0.240 |

VAS, visual analog scale; CI, confidence interval

^*^ Significant p values (P<0.05)

ANOVA Post-hoc test

**Supplementary Table 3** Post-treatment (weeks 2 and weeks 12) comparisons of results of parameters between groups

|  | | **Mean difference** | **95%CI**  **(upper and lower limits)** | **p value** |
| --- | --- | --- | --- | --- |
| **KUJALA**  **Post-treatment** | **Group 1 vs Group 2** | 15.400 | 24.013/6.786 | 0.000**^*^** |
|  | **Group 1 vs Group 3** | 13.800 | 22.413/5.186 | 0.001^*^ |
|  | **Group 2 vs Group 3** | 1.600 | 7.013/10.213 | 1.000 |
| **KUJALA**  **Post-treatment 3rd month** | **Group 1 vs Group 2** | 11.866 | 25.673/1.940 | 0.114 |
|  | **Group 1 vs Group 3** | 10.800 | 24.607/3.007 | 0.173 |
|  | **Group 2 vs Group 3** | 1.066 | 12.740/14.873 | 1.000 |
| **LEFS**  **Post-treatment** | **Group 1 vs Group 2** | 17.266 | 28.089/6.444 | 0.001^*^ |
|  | **Group 1 vs Group 3** | 15.400 | 26.222/4.577 | 0.003^*^ |
|  | **Group 2 vs Group 3** | 1.866 | 8.956/12.689 | 1.000 |
| **LEFS**  **Post-treatment 3rd month** | **Group 1 vs Group 2** | 14.400 | 23.208/5.591 | 0.001^*^ |
|  | **Group 1 vs Group 3** | 14.866 | 23.675/6.057 | 0.000^*^ |
|  | **Group 2 vs Group 3** | 0.466 | 9.275/8.342 | 1.000 |
| **TUG**  **Post-treatment** | **Group 1 vs Group 2** | 1.348 | 0.085/2.782 | 0.071 |
|  | **Group 1 vs Group 3** | 1.596 | 0.163/3.030 | 0.024 |
|  | **Group 2 vs Group 3** | 0.248 | 1.185/1.681 | 1.000 |
| **TUG**  **Post-treatment 3^rd^ month** | **Group 1 vs Group 2** | 1.743 | 0.655/2.831 | 0.001^*^ |
|  | **Group 1 vs Group 3** | 1.946 | 0.857/3.034 | 0.000^*^ |
|  | **Group 2 vs Group 3** | 0.202 | 0.885/1.290 | 1.000 |

LEFS, lower extremity functional scale; TUG, timed up and go test; CI confidence interval

^*^ Significant p values (P<0.05)

ANOVA Post-hoc test
